# Effects of HIIT training and HIIT combined with circuit resistance training on measures of physical fitness, miRNA expression, and metabolic risk factors in overweight/obese middle-aged women

**DOI:** 10.1186/s13102-024-00904-7

**Published:** 2024-05-29

**Authors:** Zhaleh Pashaei, Abbas Malandish, Shahriar Alipour, Afshar Jafari, Ismail Laher, Anthony C. Hackney, Katsuhiko Suzuki, Urs Granacher, Ayoub Saeidi, Hassane Zouhal

**Affiliations:** 1 Department of Exercise Physiology, Faculty of Sport Sciences, University of Tabriz, Tabriz, Iran; 2 Department of Exercise Physiology, Faculty of Sport Sciences, Urmia University, Urmia, Iran; 3 Department of Biochemistry, Faculty of Medicine, Urmia University of Medical Sciences, Urmia, Iran; 4 Department of Biological Sciences in sports, Faculty of Sport Sciences and Health, Shahid Beheshti University, Tehran, Iran; 5 Department of Anesthesiology, Pharmacology, and Therapeutics, Faculty of Medicine, University of British Columbia, Vancouver, Canada; 6 Department of Exercise & Sport Science, Department of Nutrition, University of North Carolina, Chapel Hill, NC USA; 7 Faculty of Sport Sciences, Waseda University, Tokorozawa, 359-1192 Japan; 8 Department of Sport and Sport Science, Exercise and Human Movement Science, University of Freiburg, Freiburg, Germany; 9 Department of Physical Education and Sport Sciences, Faculty of Humanities and Social Sciences, University of Kurdistan, Sanandaj, Kurdistan Iran; 10 Movement, Sport, Health and Sciences laboratory (M2S). UFR-STAPS, University of Rennes 2-ENS Cachan, Av. Charles Tillon, Rennes Cedex, 35044 France; 11 Institut International des Sciences du Sport (2IS), Irodouer, 35850 France

**Keywords:** High-intensity interval training, Combined training, MicroRNAs, Obesity

## Abstract

**Objective:**

The purpose of this study was to examine the effects of 10 weeks of high-intensity interval training (HIIT) and HIIT combined with circuit resistance training (HCRT) on selected measures of physical fitness, the expression of miR-9, -15a, -34a, -145, and − 155 as well as metabolic risk factors including lipid profiles and insulin resistance in middle-aged overweight/obese women.

**Methods:**

Twenty-seven overweight/obese women aged 35–50 yrs. were randomized to HIIT (*n* = 14) or HCRT (*n* = 13) groups. The HIIT group performed running exercises (5 reps x 4 min per session) with active recovery between repetitions for 10 weeks with 5 weekly sessions. The HCRT group performed 10 weeks of HIIT and resistance training with 3 weekly HIIT sessions and 2 weekly HCRT sessions. Anthropometric measures (e.g., body mass), selected components of physical fitness (cardiovascular fitness, muscle strength), levels of miRNAs (miR-9, -15a, -34a, -145, and − 155), lipid profiles (total cholesterol; TC, Triglycerides; TG, low-density lipoprotein cholesterol; LDL-C and high-density lipoprotein cholesterol; HDL-C), and insulin resistance; HOMA-IR index, were measured at baseline and week 10.

**Results:**

An ANOVA analysis indicated no significant group by time interactions (*p* > 0.05) for all anthropometric measures, and maximum oxygen consumption (VO_2max_). A significant group by time interaction, however, was found for the one-repetition maximum (IRM; p < 0.001, ES= 0.751 , moderate). A post-hoc test indicated an increase in the pre-to-post mean 1RM for HCRT (*p* = 0.001, ES = 1.83, large). There was a significant group by time interaction for miR-155 (*p* = 0.05, ES = 0.014, trivial). Levels for miR-155 underwent pre-to-post HIIT increases (*p* = 0.045, ES = 1.232, large). Moreover, there were also significant group by time interactions for TC (*p* = 0.035, ES = 0.187, trivial), TG (*p* < 0.001, ES = 0.586, small), LDL-C (*p* = 0.029, ES = 0.200, small) and HDL-C (*p* = 0.009, ES = 0.273, small). Post-hoc tests indicated pre-post HCRT decreases for TC (*p* = 0.001, ES = 1.44, large) and HDL-C (*p* = 0.001, ES = 1.407, large). HIIT caused pre-to-post decreases in TG (*p* = 0.001, ES = 0.599, small), and LDL-C (*p* = 0.001, ES = 0.926, moderate).

**Conclusions:**

Both training regimes did not improve cardiovascular fitness. But, HCRT improved lower/upper limb muscle strength, and HIIT resulted in an increase in miR-155 expression in peripheral blood mononuclear cells. Furthermore, HIIT and HCRT each improved selected metabolic risk factors including lipid profiles and glucose and insulin metabolism in overweight/obese middle-aged women.

**Trial registration:**

OSF, October, 4th 2023. Registration DOI: 10.17605/OSF.IO/UZ92E. osf.io/tc5ky. “Retrospectively registered”.

**Supplementary Information:**

The online version contains supplementary material available at 10.1186/s13102-024-00904-7.

## Introduction

Overweight/obesity is a serious health problem and its prevalence has increased rapidly during the last three decades [1], with more than a billion people worldwide being overweight or obese [2]. The global prevalence of obesity/ overweight is predicted to be 18% in men and 21% in women by 2025 [3]. Obesity is a multifactorial and complex metabolic disease with epigenetic interactions. Some of the epigenetic changes due to a sedentary lifestyle and high-caloric food lead to obesity and obesity-related disorders [4]. More recently researchers have focused on the role of microRNAs (miRNA) in obesity-related metabolic disorders. MiRNAs are 19–24 nucleotide non-coding RNA molecules involved in the posttranscriptional regulation of gene expression and as a result regulate biological processes such as metabolic risk factors [5]. Obesity affects the expression of miRNAs, which mediate diabetic macro- and microvascular complications. These changes provide unique molecular and cellular insights into their roles in obesity, insulin resistance, and diabetes mellitus [6]. In this context, miR-9, miR-15a, miR-34a, miR-145, and miR-155 are miRNAs that are associated with lipid and glucose disorders [7–10]. Decreases in miRNA expression patterns of circulating and peripheral blood mononuclear cells (PBMCs) such as miR-15, -34, -145, and − 155 are associated with pathophysiological mechanisms involved in metabolic disorders such as obesity, type 2 diabetes (T2D), and atherosclerosis [11–13].

Physical exercise has the potential to improve health and reduce metabolic disorders [14]. One of the mechanisms suggested to explain exercise-mediated regulation of cellular homeostasis is through the modulation of the miRNA expression profile [15]. There is evidence that high-intensity interval training (HIIT) improves metabolic adaptations, and HIIT combined with resistance training improves lipid profiles and decreases fasting glucose, insulin, and insulin resistance in overweight or obese women [16–18]. However, according to the recommendations of the American Sports Medicine Association for middle-aged people, combined training (endurance and resistance) is more effective in maintaining or improving the health of overweight/obese individuals, suggesting that this training method has beneficial effects on the metabolic status by increasing muscle mass [16, 18–20].

The effects of aerobic and resistance training on miRNA levels vary with the exercise type under investigation [15]. There is evidence that aerobic and resistance training have the potential to induce changes in miRNA levels in cardiac muscle, skeletal muscle, and plasma in animals (rats), untrained males, and elite male athletes [15, 21, 22]. However, the role of miRNAs and their effects on metabolic risk factors such as lipid profiles, and insulin resistance are not fully understood in PBMCs during HIIT and resistance training in overweight/obese individuals. Recent studies suggest that miRNAs are involved in intracellular interactions and changes in the expression of miRNAs can occur in response to physical exercise [15]. Therefore, we aimed to examine the effects of HIIT versus combined HIIT with circuit resistance training (HCRT) on anthropometrics (e.g., body mass index; BMI), physical fitness (e.g., maximal oxygen consumption [VO_2_max] and lower/upper limbs one-repetition maximum [1RM]) levels of PBMCs’ miRNAs (miR-9, -15a, -34a, -145, and − 155) and serum lipid profiles (total cholesterol/TC, Triglycerides/TG, low-density lipoprotein cholesterol/LDL-C and high-density lipoprotein cholesterol/HDL-C), and insulin resistance/HOMA-IR index of overweight/obese women. Based on the relevant literature [15, 21, 22], we hypothesized that particularly HCRT would induce favorable effects on anthropometrics, physical fitness, miRNAs, lipid profiles, and the insulin resistance/HOMA-IR index of overweight/obese women. In the ongoing text and for brevity, we will refer to HIIT in combination with HCRT as HCRT only.

## Materials and methods

### Participants

**Fig. 1:**
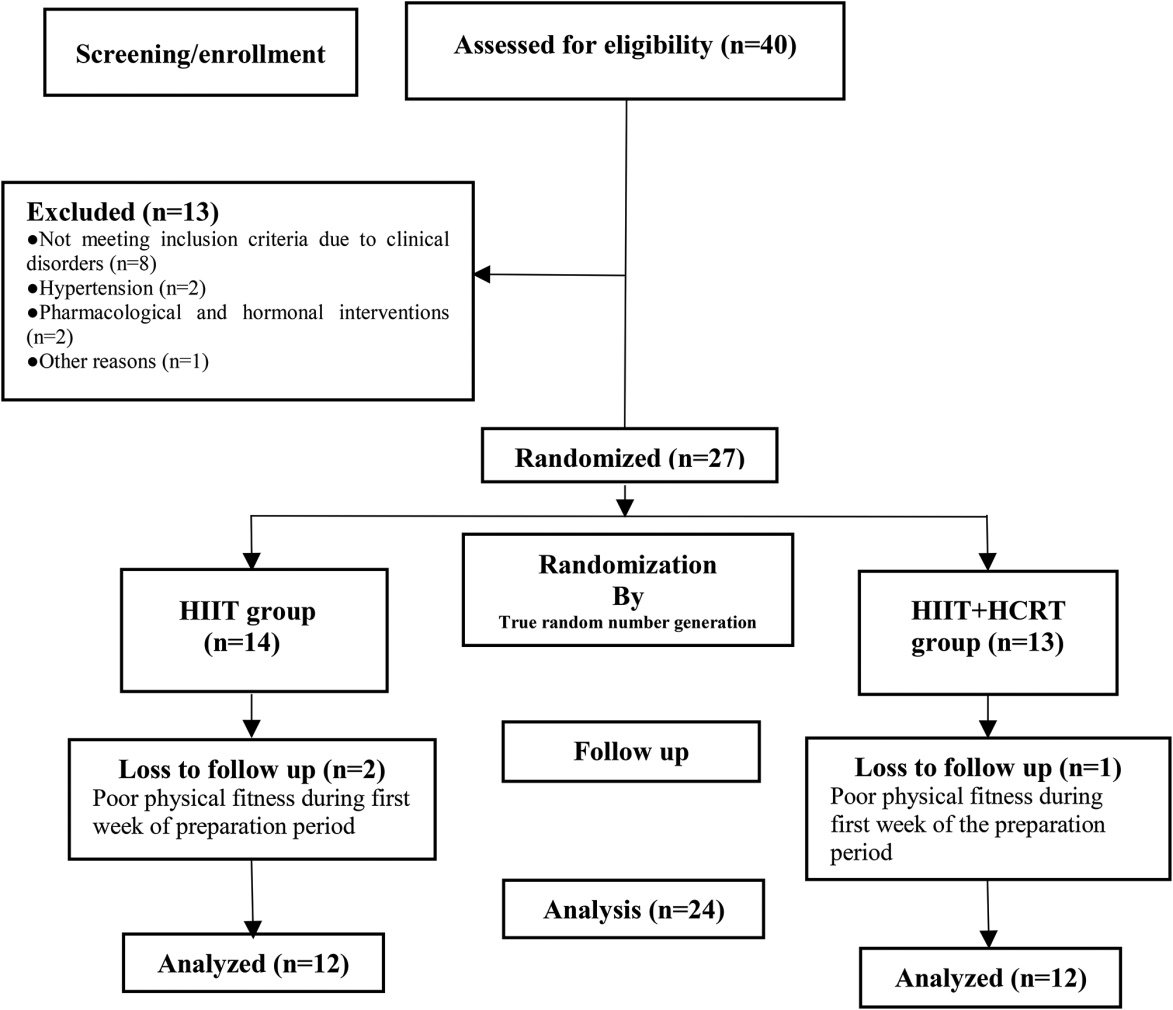
Follow-up diagram of study participants

A minimum sample size of 18 was estimated from an a priori statistical power analysis using G*Power (Version 3.1, University of Düsseldorf, Germany) [23]. The analysis revealed that this sample size would be sufficient to achieve medium-sized group-by-time interaction effects. Therefore, in order to account for potential loss to attrition, we recruited a total of *N* = 27 participants. The power analysis was computed with an assumed power of 0.80, an alpha level of 0.05, and a moderate ES (Cohen’s d) based on the outcome (i.e., % body fat) of a study with similar study design [17]. This study was approved by the regional research ethics committee of Tabriz University of Medical Sciences, Tabriz, Iran. Twenty-seven sedentary overweight/obese women volunteered to participate in this study (Fig. 1). Inclusion criteria of participants comprised: (1) females aged 35 to 50 years, (2) BMI > 25 kg/m^2^, no history of CVDs or other diseases such as metabolic disorders, diabetes mellitus, hypertension, thyroid abnormalities, fatty liver disease, pulmonary disease, musculoskeletal disease, gastrointestinal disease, polycystic syndrome, and autoimmune and neurologic diseases, (3) no history of regular exercise training, dietary regimen for body mass loss or mass gain, pharmacological and hormonal interventions and smoking for at least 6 months before the start of the study, and (4) being in the 8th to the 16th day of their menstrual cycle. Numerous studies indicate that changes in miRNAs, such as miR-155, miR-145, and miR-34a, are related to changes in the levels of estrogen and progesterone hormones [24–26]. We attempted to enroll participants who were in similar menstrual phases/periods to eliminate the potential effects of sex hormones changes on miRNAs.

Exclusion criteria of participants included (1) those who discontinued regular HIIT and HCRT protocols and/or routine dietary habits for the 10 weeks of the study, (2) non-participation in blood sampling at baseline or week-10, (3) failure to complete 80% of the scheduled physical exercise sessions (Table 1). Physical activity levels and energy expenditures of participants were expressed as metabolic equivalents of tasks (METs) using self-reported physical activity patterns. The current study was approved by the ethics committee (approval number: 1396.485) and adheres to CONSORT guidelines.

**Table 1:** Implementation of training protocols

Training group	Training period	Week number	Training intensity during sessions							% of regular participation of individuals in training sessions	Number of individuals participating in all training sessions	Compliance with each program
			Saturday	Sunday	Monday	Tuesday	Wednesday	Thursday	Friday			
HIIT group	Preparation period	1	60–65%	65–70%	Rest	70–75%	75–80%	75–80%	Rest	100%	20	90%
		2	75–80%	75–80%	Rest	75–80%	75–80%	75–80%	Rest			95%
	Main training period	3 to 10	80–85%	80–85%	Rest	80–85%	80–85%	80–85%	Rest	98%	11	93%
HCRT group	Preparation period	1	60–65%	RT 50–60%	Rest	65–70%	70–75%	RT 50–60%	Rest	100%	20	95%
		2	75–80%	RT 50–60%	Rest	75–80%	75–80%	RT 50–60%	Rest			95%
	Main training period	3	80–85%	RT 65–70%	Rest	80–85%	80–85%	RT 65–70%	Rest	98%	11	95%
		4 to 10	80–85%	RT 75–80%	Rest	80–85%	80–85%	RT 75–80%	Rest			98-100%

Note. HIIT: high-intensity interval training, HCRT: HIIT combined with resistance training (RT). Training intensity is expressed as a percentage of heart rate reserve (HRR).

All participants attended the exercise venue and received information about the protocols, procedures, benefits, and possible risks related to the study design. Written informed consent was obtained from all participants. The participants were randomly assigned to HIIT (*n* = 14) and HCRT (*n* = 13) groups (Fig. 1). True random number generation was used for study randomization, with the sealed envelope method containing the group assignment opened by the study exercise physiologists. The sample size was designed to detect a difference in study variables, with a 95% confidence interval (CI) and 80% or greater power value. Finally, three subjects (HIIT: *n* = 2 due to poor physical fitness; HCRT: *n* = 1 due to poor awareness of the study requirements) were unable to complete the study (Fig. 1). The HIIT group performed 10 weeks of high-intensity running and the HCRT group performed 10 weeks of HIIT in combination with individual circuit resistance training. Both training protocols were performed under supervision in a gymnasium during the afternoon hours.

### Exercise protocols

#### High-intensity interval training (HIIT)

**Fig. 2:**
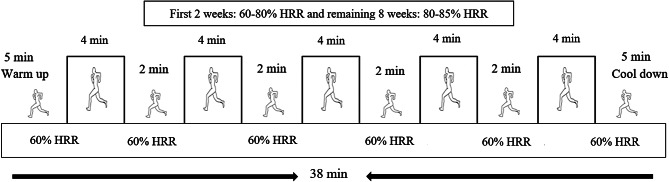
High-intensity interval training protocol (5 × 4 min) for the HIIT group (10 week). HRR = heart rate reserve

Training protocols were conducted based on the latest exercise training instructions and the training protocols for both training groups have been used from the study protocol proposed by Ramírez-Vélez et al. [27]. The HIIT group trained for 10 weeks (Fig. 2). Each HIIT session lasted 28 min and included a 5 min warm up, and consisted of 5 running intervals at an intensity of 60–80% of the heart rate reserve (HRR) for the first 2 weeks of preparatory training and 80–85% of HRR during the remaining 8 weeks, each lasting 4 min. The work to rest ratio between intervals was 2:1. The active recovery comprised running at an intensity of 60% HRR. After HIIT, a 5 min cool down consisting of running at 60% HRR combined with stretching exercises was scheduled (Fig. 2). HIIT was performed indoor (gym) at a temperature of 25–27^o^ C and relative humidity of 45–48%.

The HRR was calculated using the Karvonen formula [28]. Heart rates were recorded using a heart rate monitor (Polar Pacer, Lake Success, NY, USA) to control exercise intensity.

#### HIIT combined with circuit resistance training (HCRT)

The HCRT group performed 10 weeks of HIIT in combination with resistance circuit training. HIIT combined with HCRT was similar as with the single-mode HIIT group and was conducted three times per week. Resistance circuit training was scheduled with two sessions per week so that training volume was similar between groups. During the circuit resistance training, HCRT participants performed eight exercises for the lower and upper limbs including the leg press, knee extension/flexion, calf raise, bench press and inclined bench press, seated rowing, and lat pull-down. The training intensity was set at 50–60% of the 1RM for the first two preparatory weeks and 65–80% of the 1RM during the remaining 8 weeks. Participants performed three sets of 10–12 repetitions per exercise. Resting periods between sets and stations were 90 s and 120 s, respectively. A warm-up set was performed at 50% of the 1RM before each exercise was performed.

### Tests

#### Anthropometric characteristics

Participants’ anthropometric characteristics included the assessment of height, body mass, BMI, waist-to-hip ratio [WHR], and body fat percentage (BF%). Data were recorded using a digital wall-meter (Seca, China), a digital scale (Beurer BF800, Germany), flexible but non-stretchable tape meter and caliper, respectively. Body density was estimated using the Jackson and Pollack three-point skinfold (i.e., triceps, suprailiac, and thigh with Lange Skinfold Caliper, Cambridge Scientific Industries, Cambridge, MD, USA) equation [29], and body fat percentage was determined using the appropriate body density equation [30]. Baseline anthropometric data were collected 48 h before the first training session and after the 10th week (post – test) of the exercise protocol (48 h after the last training session) (Table 2).

**Table 2:** Physiological characteristics, miRNAs, and serum levels of cardiovascular disease risk factors of overweight/obese women before and after 10 weeks in the HIIT and HIIT + HCRT groups

Variable	HIIT group *n* = 12			HCRT group *n* = 12			Group		Time		Group by Time	
	Pre	Post	ES	Pre	Post	ES	*p*	ES	*p*	ES	*p*	ES
**Anthropometrics and selected components of physical fitness**												
Mass (kg)	72.46 ± 8.4	72.04 ± 8.1	0.05	74.21 ± 7.9	73.16 ± 8.1	0.131	0.669	0.008	0.015	0.241	0.266	0.056
BMI (kg/m^2^)	29.5 ± 3.7	29.4 ± 3.6	0.028	30 ± 2.5	29.5 ± 2.5	0.171	0.788	0.003	0.035	0.186	0.173	0.083
VO_2max_ (ml/kg/min)	30.58 ± 9.85	43.47 ± 6.16	1.61	33.72 ± 7.86	44.29 ± 8.47	1.295	0.527	0.018	< 0.001	0.787	0.382	0.035
Mean 1-RM (kg)	27.19 ± 2.41	29.85 ± 2.78	1.025	29.45 ± 5.59	40.24 ± 5.93	1.83	0.002	0.361	< 0.001	0.892	0.000	0.751
BF%	42.99 ± 3.4	35.47 ± 3.5	2.201	43.7 ± 2.5	36.9 ± 3.4	2.282	0.349	0.040	< 0.001	0.848	0.583	0.014
Mets h/week	9.01 ± 2.96	12.01 ± 2.21	1.159	10.06 ± 1.63	13.32 ± 2.01	1.793	0.177	0.081	< 0.001	0.769	0.724	0.006
**Serum levels of cardiovascular risk factors**												
TC (mg/dl)	199.0 ± 10.37	182.0 ± 27.39	0.969	200.58 ± 9.92	171.17 ± 30.93	1.44	0.021	0.220	0.001	0.415	0.035	0.187
TG (mg/dl)	168.42 ± 13.03	160.42 ± 13.67	0.599	168.92 ± 11.62	147.00 ± 10.62	1.971	0.198	0.074	< 0.001	0.867	< 0.001	0.586
HDL –C (mg/dl)	32.17 ± 5.06	34.33 ± 5.26	0.419	33.83 ± 4.02	40.75 ± 5.82	1.407	0.045	0.170	< 0.001	0.579	0.009	0.273
LDL –C (mg/dl)	137.57 ± 10.4	128.42 ± 9.35	0.926	132.93 ± 9.55	101.02 ± 33.67	1.477	0.014	0.247	< 0.001	0.448	0.029	0.200
Fasting Glucose (mmol/l)	97.83 ± 3.76	94.75 ± 3.84	0.811	98.75 ± 4.96	95.83 ± 4.64	0.607	0.546	0.017	< 0.001	0.468	0.904	0.001
Fasting Insulin (μ/ml)	4.96 ± 1.71	4.06 ± 1.56	0.550	4.03 ± 1.94	2.96 ± 1.62	0.605	0.131	0.100	0.001	0.411	0.721	0.006
HOMA-IR	1.34 ± 0.33	1.01 ± 0.32	1.031	1.34 ± 0.33	1.02 ± 0.29	1.099	0.944	< 0.001	< 0.001	0.560	0.995	< 0.001
**miRNAs**												
miR-9	1.423 ± 3.07	7.329 ± 15.62	0.673	1.725 ± 2.48	4.367 ± 4.90	0.705	0.467	0.024	0.023	0.213	1.000	< 0.001
miR-15a	1.703 ± 3.80	19.602 ± 37.10	0.602	22.134 ± 60.29	70.063 ± 160.84	0.494	0.175	0.082	0.063	0.148	0.839	0.002
miR-34a	1.791 ± 2.74	17.074 ± 30.86	1.007	3.790 ± 8.67	5.399 ± 9.01	0.568	0.048	0.166	0.063	0.148	0.901	0.001
miR-145	1.362 ± 1.79	12.388 ± 22.027	0.659	0.830 ± 0.75	6.936 ± 13.37	0.790	0.459	0.025	0.021	0.219	0.411	0.031
miR-155	0.549 ± 0.94	13.686 ± 17.62	1.232	1.901 ± 3.47	8.670 ± 15.70	0.855	0.520	0.019	0.001	0.395	0.577	0.014

Note. HIIT = high intensity interval training; HCRT = high intensity interval training + circuit resistance training; Eta = partial eta squared or estimates of effect sizes; Yrs: years; cm: centimeter; Kg: kilogram; BMI = body mass index, Kg. m^2^: kilogram. Square meters; VO_2max_ = maximal oxygen uptake, ml/kg/min: milliliter/kilogram/minute; 1RM = one-repetition maximum calculated by dividing the total maximal weight lifting in resistance exercises by the number of stations; BF = body fat; TC = total cholesterol; TG = triglycerides; HDL-C = high-density lipoprotein-cholesterol; LDL-C = low-density lipoprotein-cholesterol; HOMA-IR = homeostasis model assessment-insulin resistance; Values are mean ± SD.

#### Physical fitness

An incremental maximal effort exercise test was performed on a treadmill (Bruce test) to assess VO_2_max. After the warm up, the Bruce test (running on a treadmill) started at an initial speed of 2.7 km/h and a 10% incline, then the incline and speed increased in each stage (every three minutes) until test completion (i.e., exhaustion). After test completion, the following formula was used to estimate VO_2_max.

$${\rm{VO2max}}\,{\rm{ = }}\,{\rm{0}}{\rm{.03}}\,{\rm{ + }}\,\left( {{\rm{2}}{\rm{.74*time}}} \right)$$

To estimate the 1RM, three days before the testing session, the subjects participated in a familiarization session. During the first test session, each individual was instructed to perform a general warm up (walking, dynamic stretching, 10 repetitions per exercise at light load). Afterwards, the resistance load was progressively increased until the subject was able to perform nine or fewer repetitions per exercise. Two minutes of rest was allowed between each trial, and 3 min of rest was allowed between each strength exercise and the Brzyski equation was used to estimate the 1-RM [31]. Finally, the mean 1-RM was calculated across the eight exercises by dividing the total amount of lifted load by the number of repetitions (rep) performed.

$$1{\rm{ - RM}}\,{\rm{ = }}\,{\rm{100}}\,*\,{\rm{load}}\,{\rm{rep}}\,{\rm{/}}\,\left( {{\rm{102}}.{\rm{78}} - {\rm{2}}.{\rm{78}}\,*\,{\rm{rep}}} \right)$$

load rep: workload value of repetitions performance, expressed in kg.

rep: number of repetitions performed.

### Blood sampling

All participants were transported to the laboratory during the morning hours (between 07:30 and 08:30 AM) following a 12-hour overnight fast and 10-ml resting blood samples were taken in seated position at baseline (48 h before the start of the first session) and at week-10 (48 h after the last session). Half of the blood sample (5 ml) was used before and after the 10-weeks of HIIT and HCRT training to measure serum biomarkers (lipid profiles and insulin resistance). The remaining blood sample (5 ml) was mixed with an anticoagulant (ethylenediaminetetra-acetic acid, EDTA) to extract ribonucleic acid (RNA) in the miRNAs (miR-9, -15a, -34a, -145, -155 and UniSp6).

### RNA extraction from peripheral blood mononuclear cells (PBMCs) and complementary deoxyribonucleic- acid (cDNA) synthesis

PBMCs were isolated from EDTA-treated blood samples using standard Ficoll density gradient centrifugation. Samples were stored at -80 °C after 1 ml TRIzol® (Ribox- RNA extraction reagent, GeneAll Biotechnology, Dongnam-ro, Songpa-gu, Seoul) was added to them. After vortexing the blood samples, RNA extraction was carried out according to the manufacturer’s instructions.

Then, cDNAs of the five miRNAs were then prepared using the TaqMan microRNA reverse transcription kit (Thermo Fisher Scientific, MAN0012757, California, USA) according to the manufacturer’s instructions, and miRNAs were quantified by real-time reverse transcription–polymerase chain reaction (real-time RT-PCR) using SYBER green master mix (Real Q Plus 2×master, AMPLIQON, A323499, Denmark). Exogenously added UniSp6 was used as a spike-in normalization control. The real-time RT-PCR detection of miRNA was carried out using a stem-loop (STL) primer sequence (OG171106-250, Macrogen, Seoul, South Korea). Sample quality control was performed both spectrophotometrically (NanoDrop 1000, Thermo-Scientific™, Biocompare, USA) and by real-time PCR (RT-PCR). The RT-PCR reaction for each miRNA was performed using the following program: 1 μl CDNA, 5 μl master mix, 0.3 μl forward primer, 0.3 μl reverse primer, and DEPC-water to 10 μl. Reactions were run on a mic-PCR system (BMS Company, Queensland, Australia) at 95ºC for 10 min, followed by 40 cycles at 95ºC for 15s and 60ºC for 1 min. All reactions and analyses were performed in triplicate. Media expression of candidate miRNAs (Ct) relative to the reference gene (UniSp6) was calculated (ΔCt). Fold changes in the miRNAs expression were calculated using the equation 2^−ΔΔCT^ [32]. The sequence of primers used in the cDNA and RT-PCR synthesis steps is presented in Table 3.

**Table 3:** Primer sequence used in the cDNA and real-time RT-PCR

miRNAs	Primer sequence
Common	5´-GTG CAG GGT CCG AGG T-3´
MiR-155	(STL): 5´-GTC GTA TCC AGT GCA GGG TCC GAG GTA TTC GCA CTG GAT ACG ACA CCC CT-3´ Forward: 5´-CGG CGC TTA ATG CTA ATC GTG ATA G-3´
MiR-15a	(STL): 5´-GTC GTA TCC AGT GCA GGG TCC GAG GTA TTC GCA CTG GAT ACG ACT CAC AAA C-3´ Forward: 5´-GTA GCA GCA CAT AAT GGT TTG TGA-3´
MiR-145	(STL): 5´-GTC GTA TCC AGT GCA GGG TCC GAG GTA TTC GCA CTG GAT ACG ACA GGG AT-3´ Forward: 5´-CCG TCC AGT TTT CCC AGG AAT-3´
MiR-9	(STL): 5´-GTC GTA TCC AGT GCA GGG TCC GAG GTA TTC GCA CTG GAT ACG ACT CAT ACA-3´ Forward: 5´-CGT CTT TGG TTA TCT AGC TGT ATG AGT-3´
MiR-34a	(STL): 5´-GTC GTA TCC AGT GCA GGG TCC GAG GTA TTC GCA CTG GAT ACG ACT ACA ACC A-3´ Forward: 5´-TGG CAG TGT CTT AGC TGG TTG TA-3´
U6	(STL): 5´-GTC GTA TCC AGT GCA GGG TCC GAG GTA TTC GCA CTG GAT ACG ACA AAA ATA T-3´ Forward: 5´-GCT TCG GCA GCA CAT ATA CTA AAA T-3´ Reverse: 5´-CGC TTC ACG AAT TTG CGT GTC AT-3´

$$\Delta {\rm{Ct = Ct}}\,{\rm{target}}\,{\rm{gene - }}\,{\rm{Ct}}\,{\rm{housekeeping}}\,{\rm{gene}}$$

$$\Delta \Delta {\rm{Ct = }}\Delta {\rm{Ct}}\,{\rm{test}}\,{\rm{sample - }}\,\Delta {\rm{Ct}}\,{\rm{control}}\,{\rm{sample}}$$

$${\rm{Relative}}\,{\rm{fold}}\,{\rm{change}}\,{\rm{in}}\,{\rm{gene}}\,{\rm{expression = }}\,{{\rm{2}}^{{\rm{ - }}\Delta \Delta {\rm{Ct}}}}$$

### Serum biomarkers

Serum lipid (TC, TG, LDL-C and HDL-C) and fasting glucose levels were measured by standard automated laboratory techniques (Hitachi-912, Roche, Italy, sensitivity of 0.5 μM). Serum fasting insulin level was measured using an enzyme-linked immunosorbent assay (ELISA) kit (ParsAzmoon, Iran, sensitivity of 0.05 ng/mL) by ELISA device (BioTek ELx808, Texas, USA). Insulin resistance was estimated by the HOMA-IR index [fasting insulin (μ/ml) × fasting glucose (mmol/l)] / 22.5.

### Dietary intake assessment

Subjects who had almost the same calorie intake were used in this study. Routine diet was controlled via food records of self-reported questionnaires [30] at the start and after week 10 of the study (Table 4). Participants were asked to maintain their normal diets during the study and individuals were instructed to consume a similar diet on each sampling day.

**Table 4:** Dietary intake differences of overweight/obese women at baseline and week-10 in the HIIT and HIIT + HCRT groups

Variable	HIIT group (*n* = 12)			HIIT + HCRT group (*n* = 12)			*p* (between groups)
	Before	After	*p* (within group)	Before	After	*p* (within group)	
Total consumed calories	2000 ± 4.27	2001 ± 7.51	*p* > 0.05	2002 ± 5.82	2002 ± 4.52	*p* > 0.05	*p* > 0.05
Protein	15%	15%	-	15%	15%	-	*p* > 0.05
Carbohydrate	55%	55%	-	55%	55%	-	*p* > 0.05
Fat	30%	30%	-	30%	30%	-	*p* > 0.05

Note. HIIT = high-intensity interval training; HIIT + HCRT = high-intensity interval training + resistance circuit training; Values are means ± SDs.

### Statistical analyses

The SPSS statistical software program (SPSS Co, version 23, Chicago IL, USA) was used for data analyses. The significance level was set at *p* < 0.05. All data were expressed as means and standard deviations (SD) after data normality were checked and confirmed using the Kolmogorov-Smirnov test. The Levene test was used to assess homogeneity of variances. Between-group differences at baseline were assessed using independent samples t-tests. A 2 (group: HIIT, HCRT) by 2 (time: pre, post) analysis of variance (ANOVA) with repeated measures was used to examine the effects of HIIT versus HCRT on selected measures of physical fitness and the expression of miRNAs and metabolic risk factors in overweight/obese middle-aged women.

In case of a significant group by time interaction, Bonferroni-adjusted and group-specific post hoc tests were calculated. Partial eta-squared (η2) was used as an effect size from ANOVA output. Within-group effect sizes were calculated using the following equation: ES = (mean post – mean pre)/SD [33]. According to Hopkins [34], ES magnitudes were considered trivial (< 0.2), small (0.2–0.6), moderate (0.6–1.2), large (1.2-2.0).

## Results

All participants received treatment as allocated. No statistically significant between group baseline differences were observed for all analyzed measures.

### Anthropometrics

The ANOVA showed significant main effects of time with decreases in body mass (*p* = 0.015, ES = 0.241, small), BMI (*p* = 0.035, ES = 0.186, trivial), and BF% (*p* < 0.001, ES = 0.848, moderate). However, no significant group by time interactions (*p* > 0.05) were observed for all anthropometric parameters (Table 2).

### Physical fitness

Our results showed significant main effects of time for mean 1-RM across the eight exercises (*p* < 0.001, ES = 0.892, moderate) and VO_2_max (*p* < 0.001, ES = 0.787, moderate). A significant group by time interaction was observed for mean 1-RM performance (*p* < 0.001, ES = 0.751, moderate) only. The post-hoc test revealed a significant pre-to-post mean 1-RM increase for HCRT (*p* = 0.001, ES = 1.83, large) (Table 2).

### MicroRNAs/miRNAs (miR-9, -15a, -34a, -145 and − 155)

**Fig. 3:**
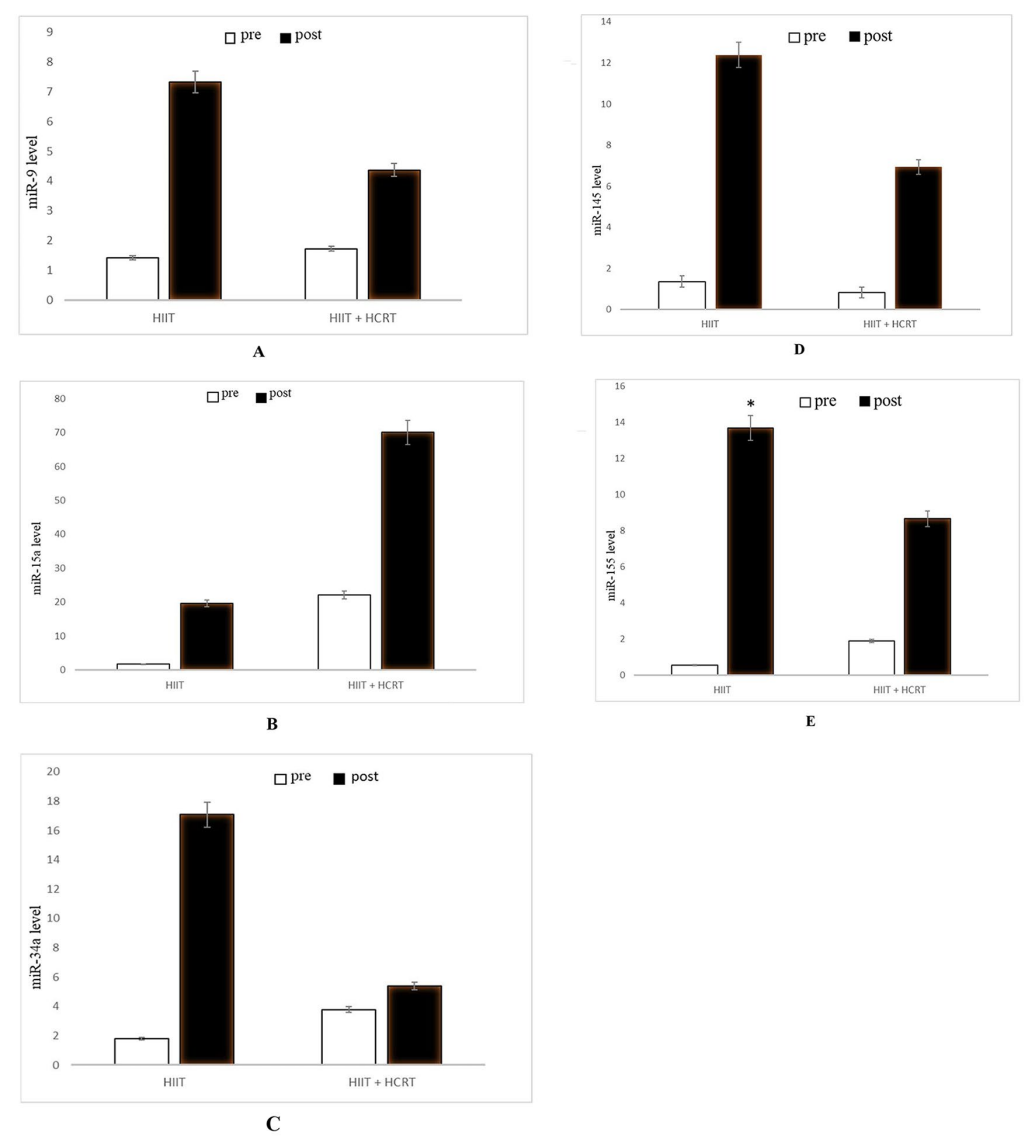
2^−∆∆Ct^ data of miRNAs following HIIT and combined training (HCRT) protocols **A**: miR-9, **B**: miR-15a, **C**: miR-34a, **D**: miR-145, **E**: miR-155 * Significantly increased in the HIIT group

ANOVA indicated a significant main effect of time for miR-155 (*p* = 0.001, ES = 0.395, small). A significant group by time interaction was found for miR-155 (*p* = 0.05, ES = 0.014, trivial). HIIT but not HCRT showed a significant pre-to-post increase for miR-155 (*p* = 0.045, ES = 1.232, large) (Table 2 and Fig. 3).

### Serum biomarkers

ANOVA showed significant main effects of time for some serum biomarkers; i.e., TC (*p* = 0.001, ES = 0.415, small), TG (*p* < 0.001, ES = 0.867, moderate), LDL (*p* < 0.001, ES = 0.448, small), insulin (*p* = 0.001, ES = 0.411, small), fasting glucose (*p* < 0.001, ES = 0.468, small), and HOMA-IR (*p* < 0.001, ES = 0.560, small) and HDL (*p* < 0.001, ES = 0.579, small).

A significant group by time interaction was observed for TC (*p* = 0.035, ES = 0.187, trivial), TG (*p* < 0.001, ES = 0.586, small), LDL-C (*p* = 0.029, ES = 0.200, small) and HDL-C (*p* = 0.009, ES = 0.273, small). Post-hoc tests revealed significant HCRT-related pre-to-post decreases for TC (*p* = 0.001, ES = 1.44, large) and HDL-C (*p* = 0.001, ES = 1.407, large). Further, HIIT resulted in significant pre-to-post decreases for TG (*p* = 0.001, ES = 0.599, small), LDL-C (*p* = 0.001, ES = 0.926, moderate) (Table 2).

## Discussion

Recent evidence suggests an important role for miRNAs as modulators of glucose and lipid metabolism by negatively regulating the expression of multiple target genes [35]. Our study demonstrated that both HIIT and HCRT training regimes failed to have a positive impact on cardiovascular fitness, although HCRT improved lower/upper limb muscle strength. Moreover, HIIT increased miR-155 expression in PBMCs. Furthermore, HIIT and HCRT improved selected metabolic risk factors including lipid profiles and glucose and insulin metabolism in overweight/obese middle-aged women.

It has previously been suggested that at least eight weeks exercise training are needed to change body composition and metabolic risk factors [36]. Some studies evaluated the effects of an HIIT program on anthropometric factors and physical fitness, where the exercise programs resulted in weight loss, improved body composition and aerobic fitness, maintained muscle mass, leading to improved muscle strength [18, 37, 38]. Moreover, the combination of HIIT with resistance training, in addition to the aforementioned effects, also stimulated energy consumption during rest by increasing muscle mass, and by lowering fat mass, muscle cells have an increased ability to alter energy metabolism during training and to regulate energy balance, which is associated with a decrease in some anthropometric variables [39].

There is evidence that physical exercise leads to a decrease in hepatic glucose production, an increase in insulin secretion from the pancreas, and a decrease in insulin resistance by improving the body fat status [40]. The increase of lipoprotein lipase (LPL) and lecithin cholesterol acyl transferase (L-CAT) enzymes, the decrease of cholesteryl ester transfer protein (CETP) and liver triglyceride lipase play important roles in changing the concentration of lipids and increasing the ability of muscle to oxidize fatty acid and reduce triglyceride [41]. A possible reason for the increase in HDL is its enhanced production by the liver following changes in LPL enzyme activity and decreases in liver lipase due to physical activity. The increase of LPL enzyme activity causes lipolysis and the release of fatty acids decomposed from TG in adipose, muscle tissue and blood circulation and in general, increases the catabolism of TG and lipoproteins rich in TG and removal of TG from the bloodstream, in this case, excess fat (free cholesterol and phospholipid) is transferred to HDL and causes its increase. On the other hand, the increase in LCAT activity caused by exercise training also feeds HDL particles. CETP is responsible for the transport of fats by HDL-c and other lipoproteins, which decreases after exercise, and the decrease in CETP leads to slower catabolism of HDL (increasing its half-life). It is possible that by reducing CETP activity due to exercise training, the conversion of HDL-c to LDL-c is reduced, thus leading to an increase in HDL-c and a decrease in LDL-c levels [41]. On the other hand, it has been reported that insulin levels decrease during and after exercise, which could change cholesterol levels [42]. This process requires increased membrane permeability to glucose, increased number of plasma membrane glucose transporters (GLUT4), increases in gene expression or activity of various proteins involved in insulin signaling, increased capillary density, increased glycogen synthetase activity in contracting muscle, and finally increased glycogen storage [43]. At the same time as insulin decreases, glucagon secretion also increases, which accelerates lipolysis [42].

The increased expression of miR-155 in our study could be due to several mechanisms. The expression of miR-155 is either decreased [44–47] or unaltered [48, 49] in breast cancer cells, serum, plasma, adipose tissue, and cerebral tissue following exercise training. The overexpression of miR-155 results in hypoglycemia, improved glucose tolerance, and enhanced insulin sensitivity in peripheral tissues, with the imbalance of miRNAs impairing glucose and lipid metabolism [50]. Similarly, a lack of miR-155 in mice also leads to hyperglycemia, glucose intolerance, and insulin resistance [51]. Lower levels of miR-155 are related to age, gender, and weight, factors that are involved in the pathogenesis of metabolic disorders [52, 53]. A lower expression of miR-155 in PBMCs from diabetic and obese non-diabetic subjects suggests a role for miR-155 in pre-diabetes [54]. The miR-155 transgene lowers serum total cholesterol and triglyceride (TG) levels, and also decreases hepatic lipid, TG, HDL and free fatty acid levels, indicating that miR-155 reduces hepatic and serum lipid levels, likely by suppressing carboxylesterase 3/triacylglycerol hydrolase (Ces3/TGH) [55] and the X receptor alpha (LXRα)-dependent lipogenic signaling pathway in the liver [56]. Thus miR-155 regulates multiple aspects of glucose and lipid metabolism by altering metabolic genes, such as by negatively regulating the expression of HDAC4, PPARγ, C/EBPβ, SOCS1, and PDK4 [57]. Moreover, C/EBPβ and HDAC4 are involved in both insulin-stimulated AKT phosphorylation and glucose uptake in hepatocytes [55].

Increased phosphorylation of AKT enhances glucose uptake and enhances glycolysis by upregulating Gck and PKM2 and downregulating PDK4 [55]. Several studies indicate that miR-155 negatively regulates PDK4 by inhibiting C/EBPβ expression, thereby improving the metabolic profile [58–63]. In addition, miR-155 enhances insulin-sensitive glucose uptake by increasing GLUT4 expression due to downregulation of HDAC4 expression. MiR-155 activates the IRS-1/PI3K/AKT insulin pathway by inhibiting SOCS1 expression [55] which reduces blood glucose and insulin sensitivity in mice [64]. Endotoxemia stimulates miR-155 expression in pancreatic β-cells, which increases insulin secretion by targeting V-maf musculoaponeurotic fibrosarcoma oncogene family protein B (Mafb) under hyperlipidemic conditions. MafB represses IL-6 expression in β-cells to inhibit GLP-1 production by islet cells. Thus, miR-155-5p improves the adaptation of β-cells to hyperlipidemic stress and compensates for obesity-induced insulin resistance to likely limit the progression of obesity and atherosclerosis [11].

Exercise can cause changes in pH, local temperature, neutrophil shear stress, and increases in cytokines and growth factors (e.g., IL-6, growth hormone), all of which can alter genomic regulation of miRNA [65]. Our study suggest that HIIT improves lipid profiles, insulin resistance, and physiological parameters due to increased miR-155 expression in overweight/obese women. Other studies reported that the hypolipidemic effects of exercise could be due to miRNA-dependent autophagy. Lipophagy is an autophagic pathway that metabolizes triglycerides, cholesterol, and other fat droplets and ultimately provides free fatty acids to produce cellular energy by mitochondrial β-oxidation [66]. The increased expression of miRNAs under conditions of cholesterol depletion alters the expression of genes involved in the metabolism of lipid and glucose. The lack of significant changes in miR-155 in the combined exercise group suggests that this mechanism should be considered with caution as it is likely that other factors can also influence changes in miR-155 levels after exercise training. In addition, the reduction of lipogenic genes occurs directly or indirectly through the lowering of insulin levels as a lipogenic hormone following exercise training, but the molecular mechanisms are not fully understood [67]. Our study indicates that the HCRT protocol had a greater effects on lipid levels and insulin resistance. However, given that HIIT increases lipolysis and improves glycemic control, our findings suggest the combination of resistance training with HIIT could lead to enhanced improvements in metabolic factors. Future research is needed to pursue this latter point.

Circulating PBMCs are affected by metabolic factors such as dyslipidemia and inflammatory molecules, and can be directly involved in obesity-related complications [68]. MiRNAs are important sources of genetic information and regulate intracellular communications [5]. Exercise alters the expression of miRNAs to cause several positive health adaptations by decreasing in obesity-related risk factors in overweight/obese [12, 69]. However, optimal levels of exercise and active muscle mass for stimulating the expression of miRNAs are currently unknown. It is possible that prolonged HCRT may be required to stimulate miRNAs expression in overweight/obese women aged 35–50 years old in our study, where HCRT caused non-significant increases in all miRNAs (miR-9, -15a, -34a, -145, and − 155). However, other molecular interactions could also reduce the effects of HCRT on miRNA expression. For example, increased miR-155 expression reduces obesity and improves lipid and glucose metabolism [53], which we also observed in the HIIT group of our study. It may be likely that more than 10 weeks of HIIT and, or HCRT could be required to cause significant increases in miRNAs (miR-9, -15a, -34a, and − 145) levels. Our novel report of increased expression of miR-155 in PBMCs of overweight/ obese women is in agreement with other findings in studies examining adipose tissue [70].

### Study limitations

Our study design included a two-group (pre-test vs. post-test) comparison of the effects of HIIT versus HCRT on measures of anthropometrics, selected components of physical fitness, miRNA expression, and metabolic risk factors. The study has a number of limitations such as the small number of participants in each group which could affect the interpretation of our results. Other limitations that should be acknowledged are; (i) the lack of a control group (i.e., we did not examine the effects of different exercise protocols alone compared to the effects of inactivity), (ii) no regulation of the diets of study subjects, (iii) an inability to regulate physical exercise outside of the study, and (iv) changes in menstrual cycle times in some participants after the 10 weeks of training (i.e., it is possible that some miRNAs may be affected by changes in sexual hormones). Collectively, these limitations suggest our findings need to be viewed with some degree of caution.

## Conclusions

Our study demonstrated that both HIIT and HCRT protocols did not improve cardiovascular fitness, but that HCRT improved lower/upper limb muscle strength. Moreover, only the HIIT program increased miR-155 expression in PBMCs. Furthermore, HIIT and HCRT each improved selected metabolic risk factors including lipid profiles and glucose and insulin metabolism in overweight/obese middle-aged women.

### Electronic supplementary material

Below is the link to the electronic supplementary material.

Supplementary Material 1

Supplementary Material 2

Supplementary Material 3

## Data Availability

The datasets generated and/or analysed during the current study are available in the Open Science Framework (OSF) repository, https://osf.io/tc5ky/.
